# CD8^+^ T-Cell Epitope Variations Suggest a Potential Antigen HLA-A2 Binding Deficiency for Spike Protein of SARS-CoV-2

**DOI:** 10.3389/fimmu.2021.764949

**Published:** 2022-01-18

**Authors:** Congling Qiu, Chanchan Xiao, Zhigang Wang, Guodong Zhu, Lipeng Mao, Xiongfei Chen, Lijuan Gao, Jieping Deng, Jun Su, Huanxing Su, Evandro Fei Fang, Zhang-Jin Zhang, Jikai Zhang, Caojun Xie, Jun Yuan, Oscar Junhong Luo, Li`an Huang, Pengcheng Wang, Guobing Chen

**Affiliations:** ^1^ Department of Neurology, Affiliated Huaqiao Hospital, Jinan University, Guangzhou, China; ^2^ Department of Microbiology and Immunology, Institute of Geriatric Immunology, School of Medicine, Jinan University, Guangzhou, China; ^3^ Guangdong-Hong Kong-Macau Great Bay Area Geroscience Joint Laboratory, Department of Microbiology and Immunology, Jinan University, Guangzhou, China; ^4^ Department of Geriatrics, Guangzhou First People’s Hospital, School of Medicine, South China University of Technology, Guangzhou, China; ^5^ Department of Primary Public Health, Guangzhou Center for Disease Control and Prevention, Guangzhou, China; ^6^ State Key Laboratory of Quality Research in Chinese Medicine, Institute of Chinese Medical Sciences, University of Macau, Macau, Macau SAR, China; ^7^ Department of Clinical Molecular Biology, University of Oslo and Akershus University Hospital, Lørenskog, Norway; ^8^ School of Chinese Medicine, Li Ka Shing (LKS) Faculty of Medicine, the University of Hong Kong, Hong Kong, Hong Kong SAR, China; ^9^ Department of Biological Products and Materia Medica, Institute of Biologics and Pharmaceuticals Research, Guangzhou, China; ^10^ Department of Systems Biomedical Sciences, School of Medicine, Jinan University, Guangzhou, China

**Keywords:** SARS-CoV-2, CD8^+^ T-cell epitope, spike protein, variations, antigen presentation deficiency

## Abstract

We identified SARS-CoV-2 specific antigen epitopes by HLA-A2 binding affinity analysis and characterized their ability to activate T cells. As the pandemic continues, variations in SARS-CoV-2 virus strains have been found in many countries. In this study, we directly assess the immune response to SARS-CoV-2 epitope variants. We first predicted potential HLA-A*02:01-restricted CD8^+^ T-cell epitopes of SARS-CoV-2. Using the T2 cell model, HLA-A*02:01-restricted T-cell epitopes were screened for their binding affinity and ability to activate T cells. Subsequently, we examined the identified epitope variations and analyzed their impact on immune response. Here, we identified specific HLA-A2-restricted T-cell epitopes in the spike protein of SARS-CoV-2. Seven epitope peptides were confirmed to bind with HLA-A*02:01 and potentially be presented by antigen-presenting cells to induce host immune responses. Tetramers containing these peptides could interact with specific CD8^+^ T cells from convalescent COVID-19 patients, and one dominant epitope (n-Sp1) was defined. These epitopes could activate and generate epitope-specific T cells *in vitro*, and those activated T cells showed cytolytic activity toward target cells. Meanwhile, n-Sp1 epitope variant *5L>F* significantly decreased the proportion of specific T-cell activation; n-Sp1 epitope *8L>V* variant showed significantly reduced binding to HLA-A*02:01 and decreased proportion of n-Sp1-specific CD8^+^ T cell, which potentially contributes to the immune escape of SARS-CoV-2. Our data indicate that the variation of a dominant epitope will cause the deficiency of HLA-A*02:01 binding and T-cell activation, which subsequently requires the formation of a new CD8^+^ T-cell immune response in COVID-19 patients.

## Introduction

COVID-19, caused by SARS-CoV-2 infection, is an emerging pandemic that is sweeping the world (1). With no highly effective clinical treatment available for COVID-19 so far, the host immune system, especially adaptive immunity, is relied on for clearance of SARS-CoV-2 (2). It has been demonstrated that both T- and B-cell clones are highly expanded in the recovery phase of COVID-19 patients (3). Besides the discovery of neutralizing antibodies (4), proliferation of CD8^+^ T cells has been observed in the lungs of patients with mild COVID-19 (5). In contrast, CD8^+^ T-cell counts were dramatically reduced in severe COVID-19 cases, and CD8^+^ T-cell exhaustion, characteristic of chronic viral infection, was detected. These findings thus revealed antigen-specific T-cell responses and indicated an important role for CD8^+^ T cells in COVID-19 recovery (6–8).

T-cell epitopes are the basis for the initiation of T-cell-mediated immune responses. Thus, identification of epitopes specific to SARS-CoV-2 and characterization of the corresponding T-cell responses are of great relevance to the understanding of COVID-19 pathogenesis and vaccine development. Computational analysis has been carried out to predict potential T-cell epitopes of SARS-CoV-2 (9), and T-cell responses specific to SARS-CoV-2 have also been tested in convalescent COVID-19 patients by using predicted peptide “megapools” (8). Recently, multiple specific CD8^+^ T-cell epitopes were identified throughout SARS-CoV-2 ORFs including spike protein (10). These epitopes were widely shared among SARS-CoV-2 isolates and located in regions of the virus that are not subject to mutational variation. Therefore, epitope identification is beneficial to the development of vaccines and medicines.

With the pandemic ongoing, variations in virus strains have been found in many countries. It was recently reported that CD8^+^ T-cell epitope variants in SARS-CoV-2 spike protein led to persistently variable SARS-CoV-2 infection with different susceptibility and severity (11). In light of this phenomenon, we analyzed the epitope variations of the spike protein among SARS-CoV-2 strains from different geographic regions and evaluated the epitope characteristics of these variations. We identified critical variations in epitope that potentially responsible for the immune escape of SARS-CoV-2.

## Results

**Figure 1:**
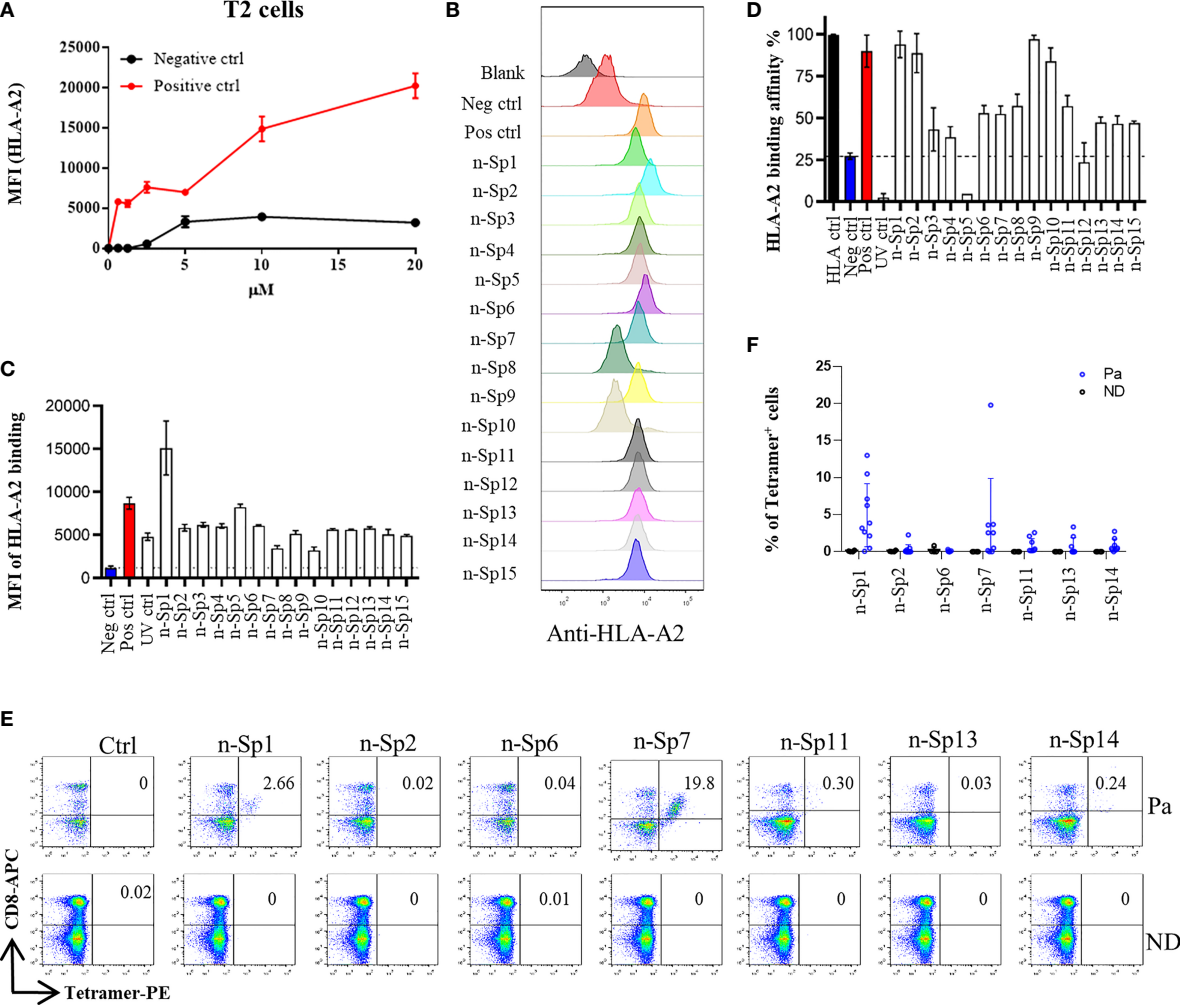
Identification of HLA-A2-restricted T-cell epitopes in SARS-CoV-2 Spike protein. Establishment of T2 binding assay. T2 cells were incubated with a series of concentrations (0 µM, 0.625 µM, 1.25 µM, 2.5 µM, 5 µM, 10 µM, and 20 µM) of positive control (Influenza A M1 peptide M58-66 GILGFVFTL) and negative control (Zika virus P30-38 GLQRLGYVL) peptides. **(A)** The stabilized HLA-A2 was detected with anti-HLA-A2 fluorescent antibody flow cytometry staining. **(B, C)** Screening of SARS-CoV-2 epitopes in T2 cells. Predicted peptides named as n-Sp1-15 were synthesized and 10 µM of each peptide was incubated with T2 cells. **(B)** The binding of peptides on T2 cells was measured with anti-HLA-A2 staining flow cytometry. **(C)** Values indicate the MFI of stabilized HLA-A2. Screening of SARS-CoV-2 epitopes with ELISA. Peptide exchange assay was performed with coated UV-sensitive peptide/MHC complex and given peptides. **(D)** The binding capability was measured by an anti-HLA-A2 ELISA assay. Blank, no peptides; Neg ctrl, negative control, peptide Zika virus peptide P30-38 GLQRLGYVL; Pos ctrl, positive control, influenza A M1 peptide M58-66 GILGFVFTL; HLA ctrl, UV-sensitive peptide without UV irradiation; UV ctrl, UV-sensitive peptide with UV irradiation. **(E, F)** Measurement of peptide-specific CD8^+^ T cells in HLA-A2^+^ convalescent COVID-19 patients and healthy donors by flow cytometry. **(E)** shows a representative FACS plot and the percentage of tetramer-positive cells is shown in **(F)** Healthy donors, *n* = 4; convalescent COVID-19 patients, *n* = 10. Ctrl: tetramer with UV-sensitive peptide; Pa, convalescent COVID-19 patients; ND, healthy donors.

To narrow down the potential candidates of SARS-CoV-2 specific antigen epitopes, we first predicted potential HLA-A*02:01-restricted CD8^+^ T-cell epitopes of SARS-CoV-2 using Immune Epitope Database and Analysis Resource (IEDB: https://www.iedb.org/), which account for the highest proportion of the Chinese population. The sequences of all the potential peptides were compared by Clustal Omega, and 15 candidate peptides specific to SARS-CoV-2 were selected (**Table 1**). To validate these predicted epitopes, we first checked whether they could be presented by HLA-A*02:01 on antigen-presenting cells (APC). The T2 cell model is an ideal model to screen HLA-A*0201-restrictive epitopes. Firstly, T2 is an APC with TAP deficiency that blocks the presentation of endogenous antigenic peptides. Secondary, it was overexpressed of HLA-A*0201, which allows the loading of MHC complexes with exogenous peptides. The binding affinity of epitopes can be inferred by the stabilization of peptide-HLA-A*0201 complexes on the cell surface. The results demonstrated that the positive control IAV epitope GIL shows an upward trend in 5 µM, 10 µM, and 20 µM, while the negative control Zika virus peptide (P30-38 GLQRLGYVL) was saturated (**Figure 1A**). Compared to the negative control Zika virus peptide (P30-38 GLQRLGYVL), all the predicted SARS-CoV-2 epitopes showed higher HLA-A2 binding affinity (**Figures 1B, C**). We further checked the direct binding of these epitopes to recombinant HLA-A*02:01 molecules (**Supplementary Figures 1A–C**). In the UV exchange peptide-MHC assay, peptides n-Sp1, n-Sp2, n-Sp9, and n-Sp10 exhibited strong binding to HLA-A*02:01 like the positive control IAV epitope GIL. However, n-Sp5 and s-Sp12 could not bind to HLA-A*02:01, while the remaining peptides showed moderate binding capability (**Figure 1D**).

**Table 1:** HLA-A*02:01-restricted peptides of S protein used in this study.

Name	Start	End	Length	Sequence	HLA	Rank	Ann_	Ann_	Smm_	Smm_	Ref
	position	position			restriction		IC50	rank	IC50	rank	
n-Sp1	2	11	10	FVFLVLLPLV	A*02	0.28	32.64	0.37	17	0.2	–
n-Sp2	133	141	9	FQFCNDPFL	A*02	1.1	9.18	0.08	56.9	1.1	–
n-Sp3	269	277	9	YLQPRTFLL	A*02	0.3	5.36	0.04	12	0.3	(10, 12)
n-Sp4	386	395	10	KLNDLCFTNV	A*02	0.42	15.27	0.14	50.06	0.7	–
n-Sp5	417	425	9	KIADYNYKL	A*02	0.7	36.12	0.4	38.82	0.7	(13)
n-Sp6	612	620	9	YQDVNCTEV	A*02	1.5	57.79	0.61	86.72	1.5	–
n-Sp7	718	726	9	FTISVTTEI	A*02	0.8	25.37	0.29	129.15	2	–
n-Sp8	733	742	10	KTSVDCTMYI	A*02	4.1	438.13	2.8	624.42	5.4	–
n-Sp9	900	909	10	MQMAYRFNGI	A*02	1.4	120.91	1.2	128.97	1.6	–
n-Sp10	1000	1008	9	RLQSLQTYV	A*02	0.7	16.66	0.17	36.74	0.7	(13, 14)
n-Sp11	1060	1068	9	VVFLHVTYV	A*02	1.2	36.56	0.4	64.88	1.2	(15)
n-Sp12	1095	1104	10	FVSNGTHWFV	A*02	0.21	13.1	0.13	21.16	0.3	–
n-Sp13	1185	1193	9	RLNEVAKNL	A*02	0.33	–	–	–	–	(14)
n-Sp14	1215	1224	10	YIWLGFIAGL	A*02	0.52	20.48	0.23	52.42	0.8	–
n-Sp15	1220	1228	9	FIAGLIAIV	A*02	0.4	10.29	0.1	20.19	0.4	(13, 16, 17)

We then prepared HLA-A*0201 tetramers with all the peptides and tested whether they could recognize antigen-specific CD8^+^ T cells from HLA-A2-positive convalescent COVID-19 patients (**Supplementary Figure 2**). Overall, 31 convalescent patients were enrolled and tested (**Table 2**), including 10 HLA-A2-positive patients. Compared to healthy donors, the antigen-specific CD8^+^ T cells were detected in convalescent patients with the tetramers of n-Sp1, 2, 6, 7, 11, 13, and 14, but not the others. Among them, n-Sp1- and n-Sp7-based pMHC tetramers demonstrated the highest proportion of antigen-specific CD8+ T cells (**Figures 1E, F**).

**Table 2:** Clinical characteristics of 31 convalescent COVID-19 patients.

Donor code	SARS CoV-2	Data of sample collection	Sex	Age	HLA-A2 restriction	Symptoms	% of tetramer+ cells						
	PCR						1	2	6	7	11	13	14
Case1	Positive	4/12/2020	Female	27	Yes	–	0.46	2.26	0	–	–	–	–
Case 2	Positive	4/12/2020	Female	25	Yes	–	10.5	0	0	0	0.17	0.7	0
Case 3	Positive	4/12/2020	Female	49	Yes	–	13	0	0.3	0	1.29	3.34	0.45
Case 4	Positive	4/12/2020	Male	54	Yes	–	–	0.47	0.07	0.49	0.14	2.05	0.24
Case 5	Positive	5/20/2020	Female	31	No	–	–	–	–	–	–	–	–
Case 6	Positive	5/20/2020	Female	38	No	–	–	–	–	–	–	–	–
Case 7	Positive	5/20/2020	Female	53	No	–	–	–	–	–	–	–	–
Case 8	Positive	6/8/2020	Male	50	No	–	–	–	–	–	–	–	–
Case 9	Positive	6/8/2020	Female	23	No	–	–	–	–	–	–	–	–
Case 10	Positive	6/17/2020	Male	35	No	–	–	–	–	–	–	–	–
Case 11	Positive	6/17/2020	Male	30	No	–	–	–	–	–	–	–	–
Case 12	Positive	6/17/2020	Male	31	No	–	–	–	–	–	–	–	–
Case 13	Positive	6/17/2020	Male	29	No	–	–	–	–	–	–	–	–
Case 14	Positive	6/17/2020	Male	35	No	–	–	–	–	–	–	–	–
Case 15	Positive	9/19/2020	Male	64	No	Mild	–	–	–	–	–	–	–
Case 16	Positive	9/19/2020	Female	54	No	Mild	–	–	–	–	–	–	–
Case 17	Positive	9/19/2020	Female	63	No	Mild	–	–	–	–	–	–	–
Case 18	Positive	10/11/2020	Female	61	Yes	Mild	0	0	0	0	2.87	0	0
Case 19	Positive	10/18/2020	Female	66	No	Mild	–	–	–	–	–	–	–
Case 20	Positive	10/18/2020	Female	57	Yes	Mild	2.13	0	0	2.55	2.58	0	1.79
Case 21	Positive	10/18/2020	Female	54	Yes	Mild	2.66	0	0.02	3.59	0.37	0.03	0.33
Case 22	Positive	10/18/2020	Female	48	Yes	Mild	7.12	0.07	0.07	3.68	2.1	0	0.87
Case 23	Positive	10/18/2020	Male	57	Yes	Mild	3.18	0.07	0	19.8	0.22	0	0.48
Case 24	Positive	10/23/2020	Female	57	Yes	Mild	3.87	0.03	0.01	2.55	0.15	0	0.24
Case 25	Positive	10/23/2020	Male	61	No	Mild	–	–	–	–	–	–	–
Case 26	Positive	11/9/2020	Female	74	No	Mild	–	–	–	–	–	–	–
Case 27	Positive	11/9/2020	Female	64	No	Mild	–	–	–	–	–	–	–
Case 28	Positive	11/9/2020	Female	49	No	Mild	–	–	–	–	–	–	–
Case 29	Positive	12/6/2020	Female	77	No	Severe	–	–	–	–	–	–	–
Case 30	Positive	12/6/2020	Male	60	No	Severe	–	–	–	–	–	–	–
Case 31	Positive	12/17/2020	Female	65	No	Severe	–	–	–	–	–	–	–

**Figure 2:**
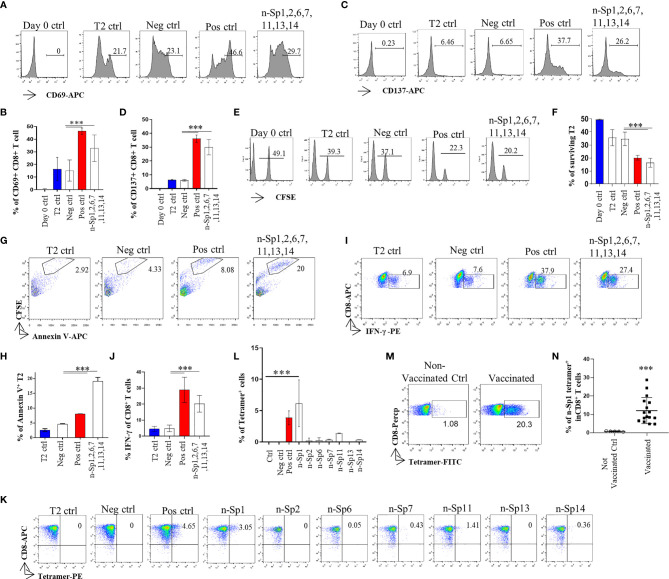
Activation of CD8^+^ T cell by peptides on SARS-CoV-2 Spike protein. **(A, B)** Mitomycin pretreated T2 cells were loaded with n-Sp1, 2, 6, 7, 11, 13, and 14 peptides and incubated with CD8^+^ T cell from healthy donors at a 1:1 ratio. Activation, cytotoxicity, and generation of epitope-specific CD8^+^ T cells were evaluated. Expression level of the T-cell activation marker CD69 was evaluated with flow cytometry after 16 h of stimulation. **(B)** Values indicate the percentage of CD69^+^CD8^+^ T cells, *n* = 3. **(C, D)** Expression level of the T-cell activation marker CD137 was evaluated with flow cytometry after 16 h stimulation. **(D)** Values indicate the percentage of CD137^+^CD8^+^ T cells. *n* = 3. **(E, F)** Epitope-specific CD8^+^ T-cell-mediated cytotoxicity was evaluated after 7 days of culture. The remaining CFSE-labeled T2 cells were calculated as survived target cells. **(F)** Values indicate the percentage of surviving T2 cells, *n* = 3. Apoptosis of T2 cells after 7 days culture. **(G, H)** Epitope-stimulated T-cell-mediated T2 apoptosis was calculated as the proportion of CFSE^+^AnnexinV^+^ cells. **(H)** Values indicate the percentage of CFSE^+^Annexin V^+^ T cells, *n* = 3. **(I, J)** The expression of IFN-γ after epitope stimulation for 7 days. IFN-γ was measured with intracellular staining flow cytometry. **(J)** Values indicate the percentage of IFN-γ^+^CD8^+^ T cells, *n* = 6. **(K, L)** Epitope-specific CD8^+^ T-cell generation after 7 days of stimulation. Stimulated CD8^+^ T cells were stained with given epitope-based tetramer and measured with flow cytometry. **(L)** Values indicate the percentage of tetramer^+^ cells, *n* = 5. Day 0 ctrl: staining before stimulation; T2 ctrl: T2 without peptide loading; Pos ctrl: positive control, T2 loaded with influenza A M1 peptide M58-66 GILGFVFTL. Neg ctrl, negative control; T2 loaded with Zika virus P30-38 GLQRLGYVL peptides. **(M, N)** A representative dot plot showing the measurement of peptide specific CD8^+^ T cells in HLA-A2^+^ non-vaccinated and vaccinated donors by flow cytometry. **(N)** Values indicate the percentage of n-Sp1 tetramer^+^CD8^+^ T cells, *n* = 5 of non-vaccinated donors and *n* = 16 of vaccinated donors. The data are represented as the mean ± SD in three independence experiments, ***p ≤ 0.001.

To further analyze whether the epitope-bound T2 cells could activate T cells, we measured the expression of T-cell activation marker CD69 to identify the proportion of peptide-specific CD8^+^ T cells after stimulation with peptide-bound T2 cells bearing peptides n-Sp1, 2, 6, 7, 11, 13, and 14. CD8^+^ T cells were isolated from HLA-A2-positive healthy donor. The results demonstrated that n-Sp1, 2, and 7 had the capability to activate CD8^+^ T cells. n-Sp1 induced the strongest response (**Figures 2A–N**). First, the expression of T-cell activation marker CD69 and CD137 increased significantly under the stimulation of the T2 cells, loaded with a peptide mixture (**Figures 2A–D**). Second, peptide-bound T2 cells stimulated T cell-mediated T2 killing, as the proportion of living T2 cells was reduced from 50% to 20.2%, which was also lower than the T2 ctrl (39.3%) and Neg ctrl (37.1%) (**Figures 2E, F**). Third, peptide-bound T2 cells stimulated T-cell-mediated T2 apoptosis as the proportion of CFSE-Annexin V^+^ T2 cells was the highest 20% (**Figures 2G, H**). IFN-γ-producing CD8^+^ T cells also increased significantly under the stimulation of the peptide-bound T2 cells (**Figures 2I, J**). Furthermore, n-Sp1-based pMHC tetramers demonstrated the highest proportion of antigen-specific CD8^+^ T cells in all the SARS-CoV-2 peptides (**Figures 2K, L**). Finally, compared with non-vaccinated donors, the proportion of n-Sp1-specific CD8^+^ T cells in vaccinated donors increased significantly (**Table 3** and **Figures 2M, N**).

**Table 3:** Clinical characteristics of SARS-CoV-2 vaccinees.

Donor	HLA-A2	Sex	Age	BMI	Time*	Symptoms
code	restriction			(kg/m^2^)	(days)	
# 1	Yes	Female	23	20.84	118	NO
# 2	Yes	Male	37	19.42	116	NO
# 3	Yes	Female	43	21.52	118	NO
# 4	Yes	Male	52	24.34	118	Local redness and swelling
# 5	Yes	Female	45	24.32	118	NO
# 6	Yes	Male	46	21.28	118	NO
# 7	Yes	Male	29	21.63	118	NO
# 8	Yes	Female	49	21.63	116	Dizziness for

						4 h
# 9	Yes	Male	23	21.34	116	NO
# 10	Yes	Female	25	22.29	118	NO
# 11	Yes	Male	34	23.43	118	NO
# 12	Yes	Male	23	21.82	116	NO
# 13	Yes	Female	38	21.43	115	NO
#14	Yes	Male	41	23.31	118	NO
# 15	Yes	Female	53	23.14	118	NO
# 16	Yes	Male	23	19..24	116	NO
# 17	Yes	Female	40	22.35	116	NO

^*^The time interval between the second injection vaccination and blood sampling.

Altogether, we therefore considered n-Sp1 to be one of the dominant CD8^+^ T-cell epitopes specific to SARS-CoV-2.

### Analysis of Immune Response in Epitope Variation of SARS-CoV-2

**Figure 3:**
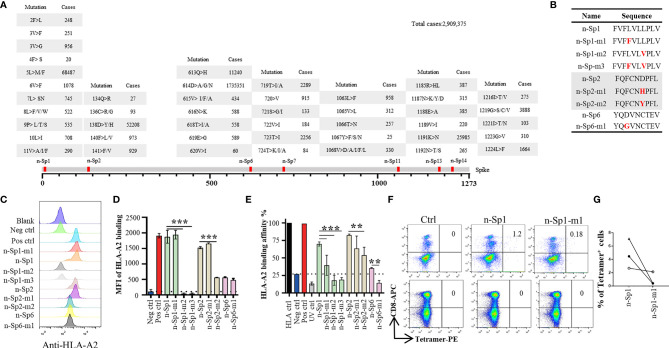
Immune response alteration in epitope mutants of SARS-CoV-2. **(A)** Variation frequency of epitopes on SARS-CoV-2 Spike protein. All SARS-CoV-2 virus sequences were collected from GISAID database, and the amino acid mutations in each epitope were calculated. **(B)** List of the epitope mutants for subsequent experiments. Comparison of epitope mutant binding to HLA-A2 in T2 cells. Wild-type and mutated epitopes were synthesized and incubated with T2 cells. **(C, D)** The peptide binding on T2 cells was measured with anti-HLA-A2 staining flow cytometry. **(D)** Values indicate the stabilized HLA-A2. Blank: no peptides; Neg ctrl, negative control; Zika virus peptide (P30-38 GLQRLGYVL), Pos ctrl: positive control; influenza A M1 peptide (M58-66 GILGFVFTL). **(E)** Comparison of epitope mutant binding to HLA-A2 by ELISA assay. Peptide exchange assay was performed with coated UV-sensitive peptide/MHC complex and given peptides. The binding capability was measured with anti-HLA-A2 ELISA assay. Blank: no peptides; HLA ctrl: UV-sensitive peptide without UV irradiation; Neg ctrl: negative control, Zika virus peptide (P30-38 GLQRLGYVL); Pos ctrl, positive control; influenza A M1 peptide (M58-66 GILGFVFTL); UV ctrl, UV-sensitive peptide with UV irradiation. **(F, G)** Measurement of n-Sp1- and n-Sp1-m1-specific CD8^+^ T cells in HLA-A2 convalescent COVID-19 patients with flow cytometry. **(G)** Values indicate the percentage of tetramer^+^ T cells, *n* = 3. Ctrl, tetramer with UV-sensitive peptide. The data are represented as the mean ± SD in three independence experiments, **p ≤ 0.01; ****p ≤ 0.001.

Phylogenetic analyses had indicated diverse variants of SARS-CoV-2 in global circulation (**Table 4**) (18), and therefore we examined the extent to which the above epitopes had been evolving in the 2,909,375 publicly available full-length SARS-CoV-2 sequences. We observed high frequency of sequence variation within these peptides, with n-Sp1, 2, 6, 7, 11, 13, and 14 bearing 19, 9, 13, 10, 12, 10, and 9 types of variations, respectively (**Figure 3A**). Among all the variations discovered, 614D>G in n-Sp6 (cases 1735351), 5L>M/F in n-Sp1 (cases 68487), and 158D>Y/D in n-Sp2 (cases 52208) were the top three most frequently observed (**Figure 3A**).

**Table 4:** The first reported mutation of SARS-CoV-2 main epitopes.

n-Sp1	n-Sp2	n-Sp6	Reported date	Country
FVFLVLLPLV	FQFCNDPFL	YQDVNCTEV	12/24/2019	China
FVFLVLLPLV	FQFCNDPFL	YQGVNCTEV	1/4/2020	Thailand
FVFLVLVPLV	FQFCNDPFL	YQDVNCTEV	1/30/2020	China-HK
FVFLVLVPLV	FQFCNYPFL	YQDVNCTEV	2/9/2020	China-HK
FVFFVLLPLV	FQFCNDPFL	YQDVNCTEV	2/10/2020	Singapore
FVFFVLLPLV	FQFCNDPFL	YQGVNCTEV	2/29/2020	UK
FVFLVLLSLV	FQFCNDPFL	YQDVNCTEV	3/2/2020	China
FVFLVLLLLV	FQFCNDPFL	YQDVNCTEV	3/10/2020	USA
FVFLVLLPLV	FQFCNHPFL	YQDVNCTEV	3/18/2020	UK
FVFLVLLPLV	FQFCNHPFL	YQGVNCTEV	3/18/2020	Russia
FVFLVLWPLV	FQFCNDPFL	YQGVNCTEV	3/18/2020	France
FVFLVLLSLV	FQFCNDPFL	YQGVNCTEV	3/24/2020	UK
FVFLVLLLLV	FQFCNDPFL	YQGVNCTEV	3/25/2020	Spain
FVFLVLLPLV	FQFCNDPFL	YQNVNCTEV	4/1/2020	UK
FVFFVLLPLV	FQFCNDPFL	YQNVNCTEV	4/2/2020	UK
FVFLVLLPLV	FQFCNYPFL	YQGVNCTEV	4/2/2020	UK
FVFLVLLTLV	FQFCNDPFL	YQGVNCTEV	4/4/2020	UK
FVFLVLLQLV	FQFCNDPFL	YQDVNCTEV	4/9/2020	India
FVFIVLLPLV	FQFCNDPFL	YQGVNCTEV	4/28/2020	USA
FVFLVLVPLV	FQFCNDPFL	YQGVNCTEV	4/29/2020	UK
FVFLVLLPLV	FQFCNYPFL	YQDVNCTEV	5/3/2020	UK
FVFLVLLPLV	FQFCNDPFL	YQSVNCTEV	5/21/2020	USA
FVFFVLLSLV	FQFCNDPFL	YQGVNCTEV	6/24/2020	UK
FVFFVLFPLV	FQFCNDPFL	YQGVNCTEV	6/26/2020	USA
FVFLVLLPLV	FQFCNDPFL	YQAVNCTEV	7/13/2020	South Korea
FVFFVLLPLV	FQFCNYPFL	YQGVNCTEV	7/18/2020	Bangladesh
FVFLVLLQLV	FQFCNDPFL	YQGVNCTEV	7/20/2020	Switzerland

To examine how these variations might affect epitope properties, we tested the binding capability of mutated epitope peptides to HLA-A*0201 through T2 binding ability and HLA-A*0201 monomer binding affinity assay (**Figures 3B–E**). For n-Sp1, the mutant FVF*F*VLVPLV (5L>F, n-Sp1-m1) showed no change in T2 stabilization, but a mild decrease in pMHC binding capability. The other mutant FVFLVL*V*PLV (8L>V, n-Sp1-m2), however, demonstrated a significant decrease in pMHC binding capability. Meanwhile, 8L>V conspicuous decrease in pMHC binding capability. The dual mutant of 5L>F and 8L>V (n-Sp1-m3) further confirmed the importance of L8 for the epitope properties of n-Sp1. Meanwhile, the mutants of n-Sp2 showed various levels of decreased pMHC binding capability, especially FQFCN*Y*PFL (138D>Y, n-Sp2-m2). In addition, the n-Sp6 mutant resulted in decreased pMHC binding capability; however, it showed similar T2 stabilization ability to the wild-type peptide (**Figures 3C–E**).

**Figure 4:**
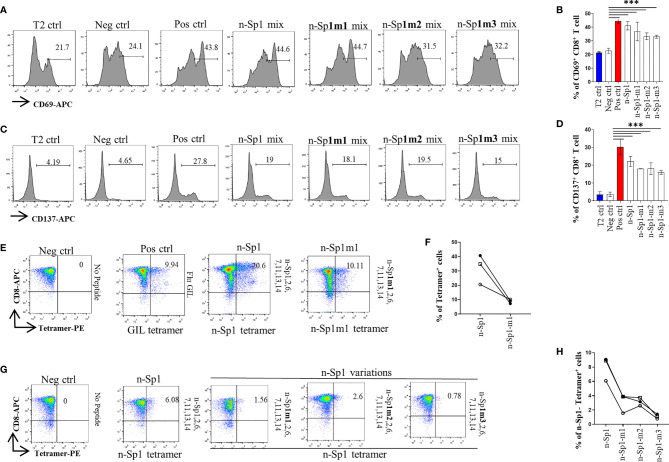
Comparison of T-cell activation and function by n-Sp1 mutants. **(A–D)** Mitomycin pretreated T2 cells were loaded with n-Sp2, 6, 7, 11, 13, 14, plus n-Sp1 (n-Sp1 mix), n-Sp1m1 (n-Sp1m1 mix), n-Sp1m2 (n-Sp2 mix), or n-Sp1m3 (n-Sp3 mix) peptides, respectively. Next, pretreated T2 cells were co-cultured with CD8^+^ T cells from healthy donors at a 1:1 ratio. Expression level of T-cell activation marker CD69 and CD137 was evaluated with flow cytometry after 16 h of stimulation. **(A, C)** are the representative plots of data in bar graphs **(B, D)**, *n* = 4. Generation of n-Sp1- and n-Sp1m1-specific CD8^+^ T cells in the same subjects. **(E, F)** The epitope-specific CD8^+^ T cells from the same subjects were measured with corresponding epitope peptide-based tetramer after 7 days of stimulation. **(E)** is the representative plot for **(F)**. **(G, H)** The epitope simulation condition and tetramer information are labeled above and below the dot plot, respectively. *n* = 3. n-Sp1 tetramer could not recognize mutant-stimulated CD8^+^ T cells in the same subjects. n-Sp1 tetramer was used to stain n-Sp1 or n-Sp1 mutant peptide-stimulated CD8^+^ T cells from the same subjects after 7 days. **(G)** is the representative plot for **(H)**. The epitope simulation condition and the tetramer information were labeled above and below the dot plot, respectively. *n* = 3. T2 ctrl: T2 cells without peptide loading; Neg ctrl: T2 cells loaded with Zika virus peptide P30-38 GLQRLGYVL; Pos ctrl: T2 cells loaded with influenza A M1 peptide M58-66 GILGFVFTL. The data are represented as the mean ± SD in three independence experiments, ***p ≤ 0.001.

By using HLA-A*0201 tetramers containing the mutated epitopes, no CD8^+^ T cells specific to n-Sp1 mutation were detected in HLA-A2-positive convalescent COVID-19 patients from Guangzhou, China (**Figures 3F, G**). However, T2 cell loaded with n-Sp1 mutants were able to increase the expression of T-cell activation marker CD69 and CD137 (**Figures 4A–D**) and generate CD8^+^ T cells specific to the mutants (**Figures 4E, F**), even though the proportion of CD8^+^ T cells specific to mutation was less than wild type in the same host (**Figure 4F**). More importantly, although n-Sp1-m1 could activate T cells, the wild-type n-Sp1 tetramer could not recognize antigen-specific CD8^+^ T cells induced by n-Sp1 mutants (**Figures 4G, H**).

Among all the variations reported so far, 614D>G was the most frequently observed (**Figure 3A**). However, n-Sp6 showed a lack of T-cell activation ability (**Supplementary Figures 3A–D**), and a low proportion of antigen-specific T cells in convalescent COVID-19 patients (**Supplementary Figures 3E, F**); therefore, it was not considered as the dominant CD8^+^ T-cell epitope.

**Figure 5:**
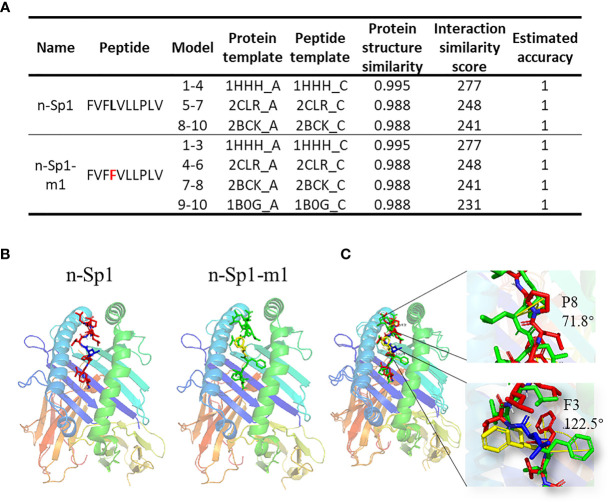
Molecular mechanism of epitope mutant presentation on HLA-A2. **(A)** GalaxyPepDock was used for molecular docking to demonstrate the pMHC structure of n-Sp1 and n-Sp1-m1. Summaries of molecular docking, including the templates used for docking. The first 4 characters of the template were PDB ID number. The models were ranked by the similarity scores and combined with the same parameters in one line. **(B)** Simulated crystal structure of pMHC were presented for n-Sp1 and n-Sp1-m1. The gray stick represented epitope peptide. **(C)** Comparison of pMHC structure between n-Sp1 and n-Sp1-m1. The HLA-A2 structure was shown as ribbon diagrams and n-Sp1 and n-Sp1-m1 mutation sites were shown as green and red sticks, respectively. The angle altered amino acids were indicated as F3 and P8 at position 4 and 8, respectively.

To further explore the mechanisms of CD8+ T-cell epitope variations’ HLA-A2 binding deficiency, GalaxyPepDock (19) was performed to compare the structure of pMHC (HLA:0201; PDB ID: 3mrb) between n-Sp1 and n-Sp1-m1 (5L>F) (**Figures 5A, B**) (19). The two benzene rings on 3F and 4F made the peptide slightly rotated, and the benzene ring on F3 bulged out from the pMHC surface, as did 8P (**Figure 5C**, and **Supplementary PDB Files**). All of these structural changes might serve as the molecular basis for the antigen presentation and CD8^+^ T-cell activation deficiency of n-Sp1-m1 mutant.

Overall, these results indicated that these emerged variations might have caused a deficiency in antigen presentation of the dominant epitopes, which then required the formation of a new CD8^+^ T cell immune response in COVID-19 patients.

## Discussion

Similar to SARS-CoV and MERS-CoV, SARS-CoV-2 has been demonstrated to induce T-cell responses to its spike protein (20). Our study identified HLA-A*02:01-restricted CD8^+^ T-cell epitopes on SARS-CoV-2 spike protein. We first predicted 15 potential HLA-A*02:01-restricted CD8+ T-cell epitopes. Among them, n-Sp11, n-Sp13, and n-Sp15 have also been reported as SARS-CoV-specific epitopes and have been shown to stimulate specific CTL responses (21, 22). In the study of Ferretti et al., memory CD8^+^ T cells from convalescent COVID-19 patients could be activated by 29 epitope peptides, among which only 3 peptides were in spike protein, including n-Sp3 (10). In our study, n-Sp3 also showed some HLA-A*02:01 binding capability, but it was much lower than the dominant epitope n-Sp1. Although their ability to activate memory CD8^+^ T cells from HLA-A2-positive convalescent COVID-19 patients were not tested, our results did show that mixed epitope-loaded antigen-presenting cells could activate T cells from healthy donors. Our study used T2 as antigen-presenting cells. In this model, a single epitope peptide loaded onto T2 cells could not activate CD8^+^ T cells from healthy donors. Notably, the proportion of n-Sp1-specific T cells produced by mixed epitope activation was even higher than that of the positive control (Influenza A M1 peptide M58-66 *GILGFVFTL*). Combining the results of HLA-A*02:01 binding capability, the proportion of antigen-specific CD8^+^ T cells in HLA-A2-positive convalescent COVID-19 patients, and the ability to activate T cells, we therefore propose that n-Sp1 is the dominant CD8^+^ T-cell epitope specific to SARS-CoV-2. Since the antibody used in our study could not distinguish the subtypes of HLA-A2, further studies were required to detect the epitopes for other subtypes of HLA-A2, such as HLA-A*02:06.

An artificial antigen-presenting cell (aAPC) system was used in this study, providing a convenient protocol to validate T-cell epitopes without the need of a high biosafety level laboratory. The T2 cell line was selected for our study for several reasons. First, its intrinsic deficiency in endogenous antigen presentation made it more reliable to evaluate the presentation of exogenous epitope by HLA-A*02:01, without the interference of endogenous epitopes. Next, it demonstrated an excellent capability to stimulate CD8^+^ T cells, with a high proportion of antigen-specific CD8^+^ T cells generated upon the primary immune challenge. Lastly, it could also be a target cell for cytolytic assays after being loaded with a given epitope. Altogether, it is an ideal model to identify T-cell epitopes for novel antigenic peptides, especially due to the low biosafety level restrictions. By using this cell model, we demonstrated that the novel epitopes from spike protein of SARS-CoV-2 had the capability to initiate CD8^+^ T-cell responses, even in unexposed donors. These epitopes might be candidates for vaccine development in the future.

Since the virus variants have been emerging geographically around the world, especially with ongoing mutation of these epitopes, it is critical to explore how the variations could affect the transmissibility and pathogenicity of the virus. Among all the variations reported so far, 614D>G and 613Q>H in n-Sp6 and 5L>F in n-Sp1 were the three most frequently observed (**Figure 3A**). Although 614D>G replacement has been reported as the dominant pandemic form in an epidemiological analysis (23), the G614 peptide-mediated T-cell activation was not significantly altered compared to the D614 peptide. The possible explanation for this phenomenon is that G614 has a stronger binding ability with Angiotensin-converting Enzyme 2 (ACE2) (24), but it is not involved in the induction of T-cell immune responses. Our result was also in accordance with the report that although the G614 variant showed higher viral loads in patients, it did not increase disease severity (25).

Although the n-Sp1 5L>F mutant could induce T-cell activation, its specific T cells were undetectable in the convalescent COVID-19 patients. A possible reason is that the patients tested here were infected with a wild-type virus strain during the early stage of the epidemic, and a new CD8^+^ T-cell immune response to the 5L>F mutant was needed. In addition, n-Sp1 8L>V mutation not only significantly reduced its binding to HLA-A*02:01, but also showed a decreased proportion of n-Sp1-specific CD8^+^ T cells, which might contribute to the immune escape of SAR-CoV-2.

Taken together, our data indicated that the variation of a dominant epitope might cause the deficiency in antigen presentation, which subsequently required the formation of a new CD8^+^ T-cell immune response in COVID-19 patients.

## Methods

### Human Subject Enrollment

The Institutional Review Board of the Affiliated Huaqiao Hospital of Jinan University approved this study. Unexposed donors and non-vaccinated donors were healthy individuals enrolled in Guangzhou Blood Center and confirmed with a negative report for SARS-CoV-2 RNA RT-PCR assay. These donors had no known history of any significant systemic diseases, including, but not limited to, hepatitis B or C, HIV, diabetes, kidney or liver diseases, malignant tumors, or autoimmune diseases. Convalescent donors included subjects who were hospitalized for COVID-19 or confirmed SARS-CoV-2 infection with RNA RT-PCR assay (**Table 2**). SARS-CoV-2 vaccinees were recruited about 4 months after vaccination with the inactivated vaccine (Beijing Institute of Biological Products of Sinopharm). We recruited a cohort of 65 SARS-CoV-2 vaccinees, including 16 who were HLA-A2 positive. All subjects provided informed consent at the time of enrollment that their samples could be used for this study (**Table 3**). All subjects provided informed consent at the time of enrollment that their samples could be used for this study. Complete blood samples were collected in acid citrate dextrose tubes and stored at room temperature prior to PBMC isolation and plasma collection. Samples’ HLA-A2 phenotype were analyzed by flow cytometry of HLA-A2 antibody staining [PE anti-human HLA-A2 (clone BB7.2) BioLegend Cat#343305;RRID : AB_1877228].

### HLA-A2-Restricted T-Cell Epitope Prediction

The spike protein sequences of SARS-CoV-2 Wuhan-Hu-1 strain (NC_045512.2), SARS-CoV GD01 strain (AY278489.2), MERS-CoV Riyadh_1_2012 strain (KF600612.1), and human coronavirus OC43 strain (KF530099.1) were used for T-cell epitope prediction with the “MHC I Binding” tool (http://tools.iedb.org/mhci). The prediction method used was IEDB Recommended 2.22 (NetMHCpan EL), with MHC allele selected as HLA-A*02:01, the most frequent class I HLA genotype among Chinese population (26, 27). The spike protein sequences of all four virus strains were aligned with Clustal Omega with SARS-CoV-2 as reference, and potential peptide candidates specific to SARS-CoV-2 were selected for further validation.

### Analysis of SARS-CoV-2 Spike Protein Variations

All available SARS-CoV-2 amino acid sequences of spike protein were collected from the GISAID database (GISAID.org). The sequences of each epitope were extracted for further analysis with Microsoft Office Excel 2010 and Graphic Prism (Version 7).

### Peptide Screening With T2 Cells

All peptides were synthesized in Genscript Biotech Co., Ltd (Nanjing). To establish the T2 binding assay, T2 cells were seeded into 96-well plates, and then incubated with peptides at 0 µM, 0.625 µM, 1.25 µM, 2.5 µM, 5 µM, 10 µM, and 20 µM at 37°C for 4 h, followed by screening of SARS-CoV-2 spike protein epitopes at 10 µM. DMSO was set as blank control, Influenza A M1 peptide (M58-66 GILGFVFTL) was set as positive control, and Zika virus peptide (P30-38 GLQRLGYVL) was set as negative control. Cells were stained with FITC anti-human HLA-A2 antibody (BioLegend, Cat#343303, San Diego, California, USA) at 4°C in the dark for 30 min and acquired in flow cytometer FACS Canto (BD).

### Expression and Purification of HLA-A2 Protein

The HLA-A2 heavy chain and β2-Microglobulin genes were cloned into the pTXBI vector through *Spe*I and *Nde*I restriction sites. Both constructs were confirmed by DNA sequencing (Shanghai Sangon Biotech, Co., Ltd). Under a heat shock at 42°C, the constructs were transformed into *E. coli* BL21, and single colonies were picked and cultured in 2 ml LB broth containing 0.1 mg ml^−1^ ampicillin at 37°C and 370 rpm for 16 h. The culture was subsequently expanded to 400 ml and induced with 0.1 mM IPTG at 24°C for 4 h. The cells were harvested and lysed, followed with 50 min sonication (work 5 s, gap 3 s) on ice. After centrifugation, the supernatant was collected into chitin resin balanced with Column Buffer (20 mM Tris-HCl, pH 8.5, 0.5 M NaCl, 1 mM EDTA, 0.05% Tween 20) at a low flow rate (0.5 ml/min). After 15 column volume washing, on-column cleavage was conducted by quickly washing the column with 3 column volumes of the Cleavage Buffer (Column Buffer supplemented with 50 mM DTT). The column was incubated at 4°C for 36 h and the target protein was eluted using 3 column volumes of Column Buffer. Samples of every step were collected for SDS-PAGE analysis.

### Preparation of Peptide-HLA-A2 Monomer

UV-sensitive peptide-HLA-A2 monomer was refolded in refolding buffer (100 mM Tris-Cl, pH 8.3, 5 M urea, 0.4 M arginine HCl, 3.7 mM cysteamine, 6.3 mM cysteamine, and 2 mM EDTA) at 4°C. In brief, 500 µl of 20 mM UV-sensitive peptide VPLRPM’J’Y [J represents Fmoc-(S)-3-amino-3-(2-nitrophenyl) propionic acid] and 30 mg of β2-Microglobulin were added to the refolding mixture *via* syringe. Next, 10 mg HLA-A2 heavy chain protein was added to the refolding mixture *via* syringe every 8 h with 3 injections and 30 mg protein in total, and let the mixture spin in the refrigerator for 15 h. The refolding mixture was then dialyzed against 20 mM Tris (pH 8.0) for 36 h, changing the buffer about every 12 h. Finally, the mixture was filtered and purified followed by purification *via* size exclusion chromatography (Superdex 75pg, GE Healthcare). Samples of every step were collected for SDS-PAGE analysis.

### HLA-A2 ELISA

Ninety-six-well U-bottomed plates were coated with 100 µl of 0.5 µg ml^−1^ streptavidin (BioLegend Cat#270302, San Diego, California, USA) at room temperature (18–25°C) for 16–18 h, washed 3 times with Washing Buffer (BioLegend Cat#421601, San Diego, California, USA), and blocked with Dilution Buffer (0.5 M Tris, pH 8.0, 1 M NaCl, 1% BSA, 0.2% Tween 20) at room temperature for 30 min. Samples were prepared by 300-fold dilution in Dilution Buffer and kept on ice until usage. DMSO was set as blank control, Influenza A M1 peptide (M58-66 GILGFVFTL) as positive control, and Zika virus peptide (GLQRLGYVL) as negative control. One hundred microliters of samples was added in duplicate and incubated for 1 h at 37°C. After washing three times with Washing Buffer, 100 µl of diluted HRP-conjugated antibodies (BioLegend Cat#280303, San Diego, California, USA) were added and incubated for 1 h at 37°C and then washed thoroughly. One hundred microliters of substrate solution (10.34 ml of DI Water, 1.2 ml of 0.1 M citric acid monohydrate/tri-Sodium citrate dihydrate, pH 4.0, 240 µl of 40 mM ABTS, and 120 µl of hydrogen peroxide solution) was added and incubated for 8 min at room temperature in the dark on a plate shaker at 400–500 rpm. The reaction was stopped with 50 µl of Stop Solution (2% w/v oxalic acid dihydrate) and absorbance at 414 nm was read using a plate reader.

### Generation of Antigen-Specific HLA-A2 Tetramer

Peptide stock solution (10 mM) was diluted to 400 µM in PBS. Twenty microliters of diluted peptide and 20 µl of 200 µg ml^−1^ UV-sensitive peptide-HLA-A2 monomer was added into 96-well plates and mixed well by pipetting up and down. The plates were sealed, spun down at 4°C, and exposed to UV light for 30 min on ice. The plates were then incubated for 30 min at 37°C in the dark. Thirty microliters of peptide-exchanged monomer and 3.3 µl of PE-streptavidin (BioLegend Cat#405203, San Diego, California, USA) were mixed in a new plate and incubated on ice in the dark for 30 min. After the incubation, 2.4 µl of blocking solution [1.6 µl 50 mM biotin (Thermo Fisher, Cat#B20656, OR, USA) and 198.4 µl PBS] was added to stop the reaction and biotinylation was done at 4–8°C overnight.

### Staining of Cell-Surface CD8 and Tetramer

PBMCs were isolated from peripheral venous blood of healthy donors and convalescent COVID-19 patients. The HLA-A2^+^ donors were identified by using flow cytometry. Briefly, 10^6^ PBMCs were stained with FITC anti-human HLA-A2 antibody at 4°C in the dark for 30 min and acquired by flow cytometer. HLA-A2-positive PBMC samples were further stained with PE-labeled tetramer (home-made) and APC-labeled human CD8 antibody (BioLegend Cat#344721, San Diego, California, USA), and acquired by flow cytometer FACS Canto (BD).

### T-Cell Activation

HLA-A2 expressing T2 cells were loaded with peptides for subsequent T-cell activation. Briefly, T2 cells were pretreated with mitomycin for 30 min to stop cell proliferation and incubated with given epitope peptides for 4 h for peptide loading. Peptide-loaded T2 cells were stained with FITC anti-human HLA-A2 antibody (BioLegend, Cat#343303, San Diego, California, USA) to analyze the loading rate.

CD8^+^ T cells were purified from PBMC with immunomagnetic negative selection (Stemcell, Cat#17953, Vancouver, BC, Canada). Briefly, PBMCs were incubated with antibody cocktail and then RapidSpheres, and then the magnet was applied and unbound CD8^+^ T cells were recovered from the supernatant. The purity of CD8^+^ T cells was checked using flow cytometry. CD8^+^ T cells (0.5 × 10^6^) from healthy donors were co-cultured with 0.5 × 10^6^ mixed peptide-loaded, CFSE-labeled T2 cells, in media containing 1 µg ml^−1^ anti-human CD28 antibodies (BioLegend Cat#302901, San Diego, California, USA) and 50 IU ml^−1^ IL-2 [SL PHARM, Recombinant Human Interleukin-2 (^125^Ala) Injection]. IL-2 (50 IU ml^−1^) and 20 µM peptides (n-Sp1, 2, 6, 7, 11, 13, and 14) were supplemented every 2 days. The T-cell activation marker CD69 (BioLegend Cat#310909, San Diego, California, USA), CD137 (BioLegend Cat#309809, San Diego, California, USA), and tetramer-specific CD8^+^ T-cell proportion were measured, along with apoptosis marker Annexin V-APC (BioLegend Cat#640919, San Diego, California, USA) on T2 cells. Parameters were evaluated after 16 h and 7 days, respectively. On day 7, cells were re-stimulated with peptides for 6 h in the presence of GolgiPlug and GolgiStop (BD Cat#550583, Bedford, NY, USA) plus 50 IU ml^−1^ IL-2, and the production of IFN-γ was checked by intracellular FACS staining with anti-IFN-γ-PE (BioLegend Cat#506507, San Diego, California, USA) (28).

### Structure Docking

To evaluate the affinity of peptides and HLA molecules, GalaxyPepDock was used for molecular docking to calculate and simulate the binding between both. Firstly, the available structure of HLA: 0201 (PDB ID: 3mrb) was downloaded from the RSCB PDB server (https://www.rcsb.org/). Next, GalaxyPepDock, a template-based docking program for peptides and proteins, which will generate 10 models to evaluate the results of the docking, was used to perform molecular docking. The top model with the highest interaction similarity score (the similarity between the template structure amino acid residues that match the target amino acid and the template amino acid) was selected and visualized using Discovery Studio 4.5 (Dassault Systèmes Biovia, San Diego, CA).

### Statistical Analysis

GraphPad Prism 7 software (version 7.0; GraphPad Software, La Jolla, California) was used for statistical analysis. The data are presented as the mean ± SD in all figures. One-way ANOVA was performed for group analysis. *p-*values less than 0.05 were statistically significant.

## Data Availability Statement

The original contributions presented in the study are included in the article/**Supplementary Material**. Further inquiries can be directed to the corresponding authors.

## Ethics Statement

The studies involving human participants were reviewed and approved by the Affiliated Huaqiao Hospital, Jinan University, Guangzhou, China. The patients/participants provided their written informed consent to participate in this study.

## Author Contributions

GC and PW designed the project. CQ and CCX performed the experiments. ZW and XC analyzed the clinical information and performed sample collection. LM performed the molecular docking. LG, JD, and JZ assisted with experiments. GZ, JS, CJX, and JY assisted with clinical information and sample collection. CQ, LH, OL, PW, and GC analyzed the data. HS, EF, and Z-JZ assisted with data analysis. CQ, PW, and GC wrote the manuscript. All authors contributed to the article and approved the submitted version.

## Funding

This work was supported by grants from the National Key Research and Development Program of China (2018YFC2002003), the Natural Science Foundation of China (U1801285 and 81971301), Guangzhou Planned Project of Science and Technology (201904010111 and 202002020039), Zhuhai Planned Project of Science and Technology (ZH22036302200067PWC), and the Initial Supporting Foundation of Jinan University.

## Conflict of Interest

The authors declare that the research was conducted in the absence of any commercial or financial relationships that could be construed as a potential conflict of interest.

## Publisher’s Note

All claims expressed in this article are solely those of the authors and do not necessarily represent those of their affiliated organizations, or those of the publisher, the editors and the reviewers. Any product that may be evaluated in this article, or claim that may be made by its manufacturer, is not guaranteed or endorsed by the publisher.
