# Supplementary figures and images for: CD8+ T-Cell Epitope Variations Suggest a Potential Antigen HLA-A2 Binding Deficiency for Spike Protein of SARS-CoV-2
Source: Front Immunol. 2022 Jan 18;12:764949. doi: 10.3389/fimmu.2021.764949 (PMC8804355; doi:10.3389/fimmu.2021.764949)

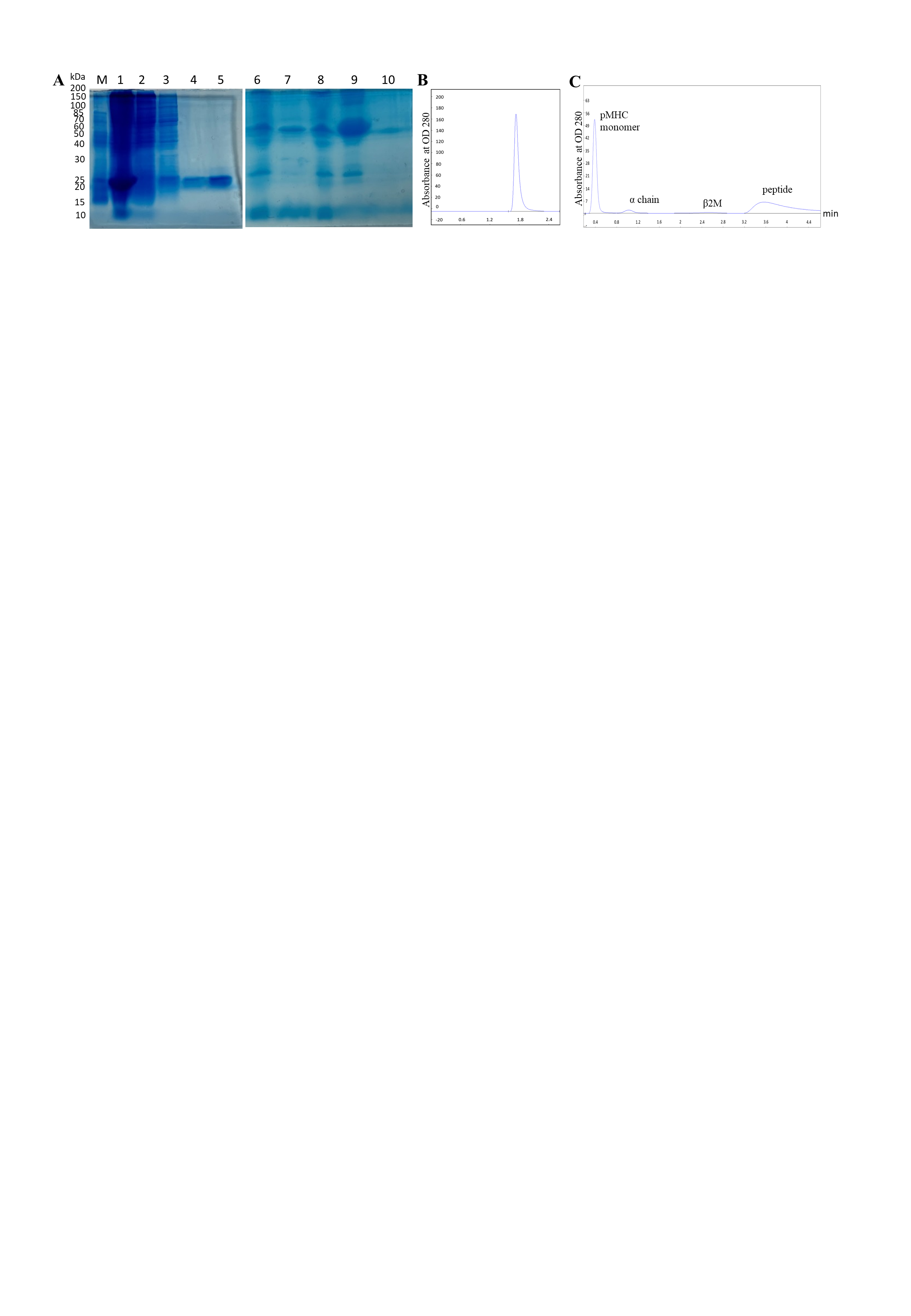

Supplement: Supplementary file 1 [file Image_1.tif]

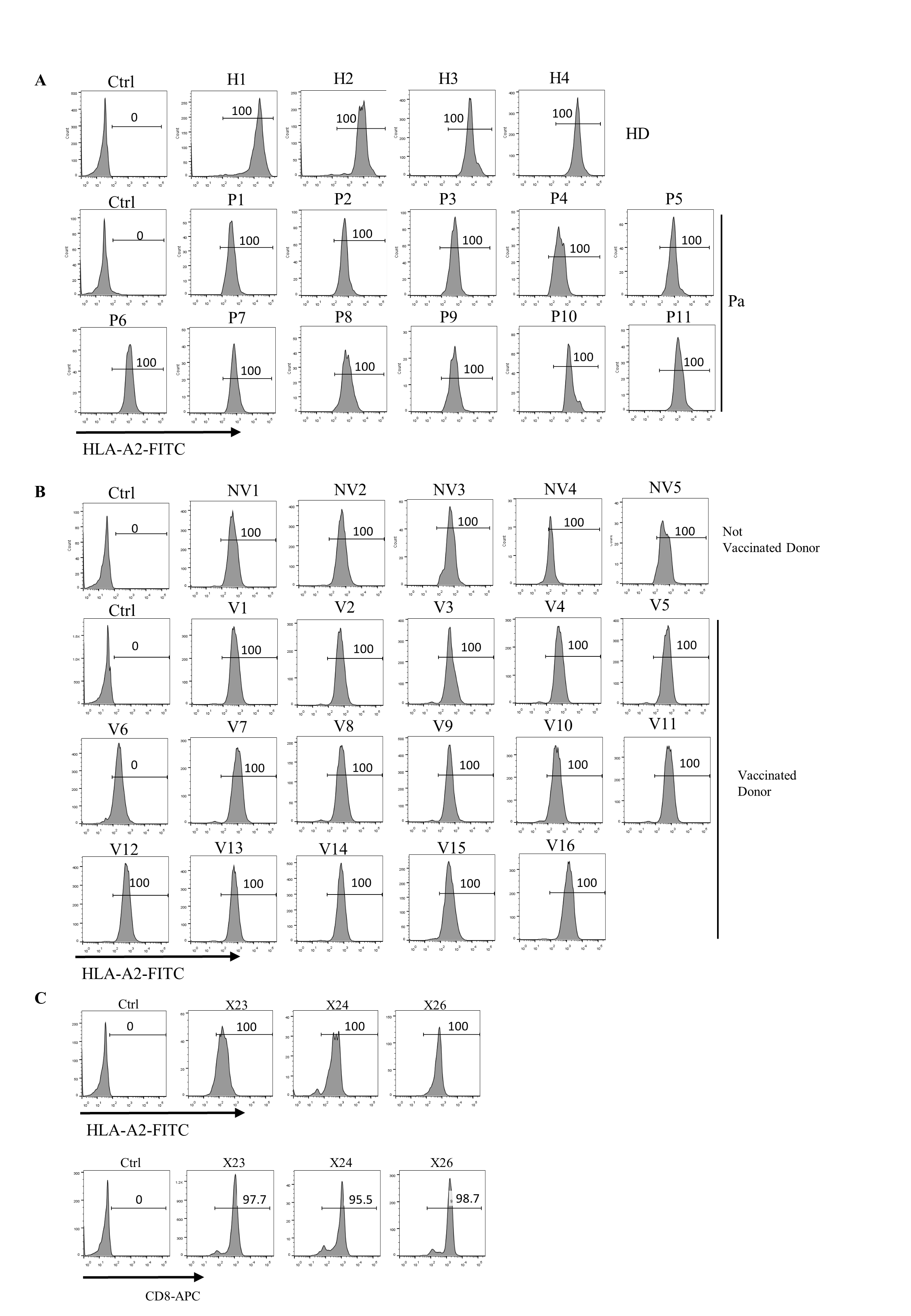

Supplement: Supplementary file 2 [file Image_2.tif]

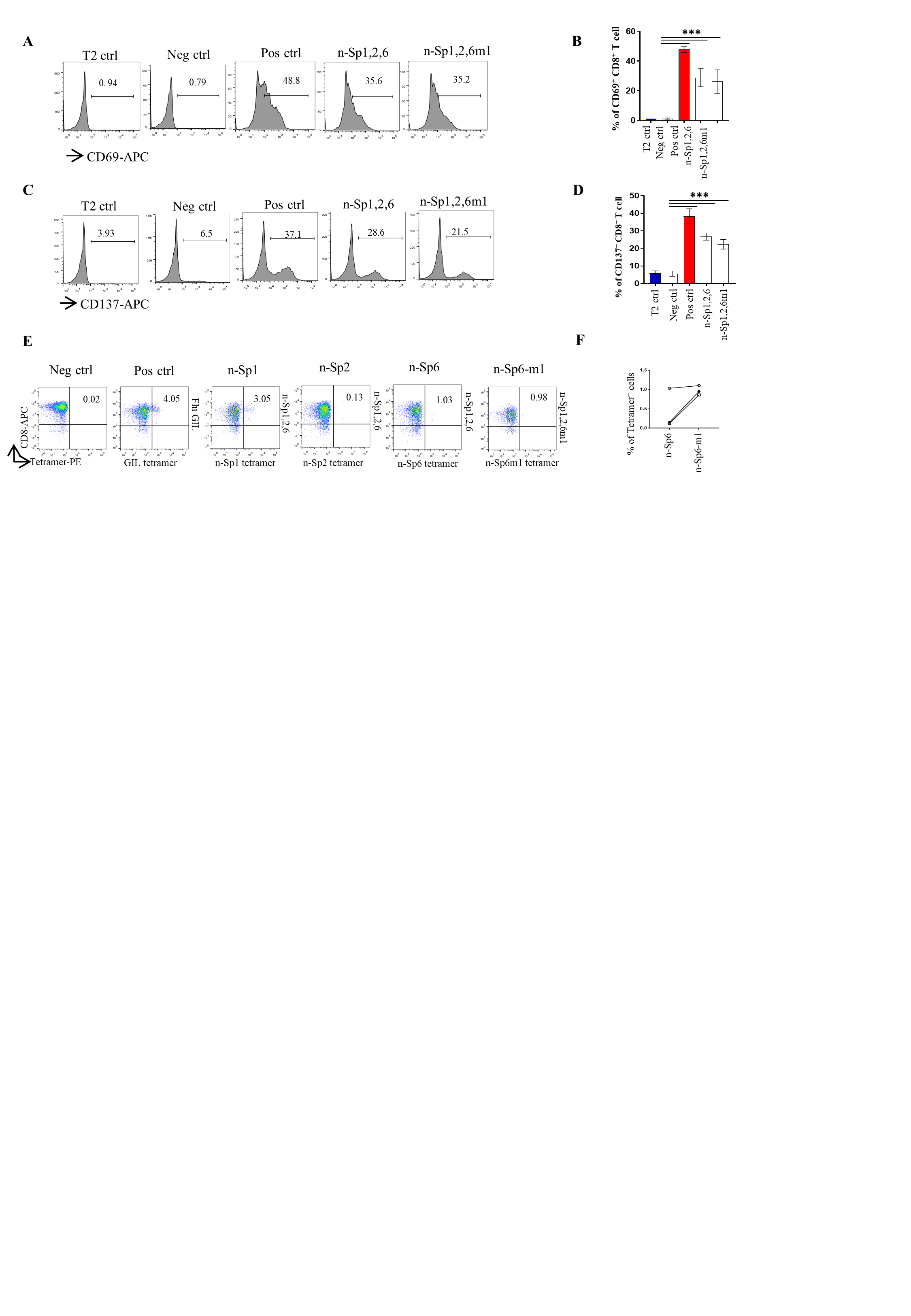

Supplement: Supplementary file 3 [file Image_3.tif]
